# Anti-inflammatory effect of a retrovirus-derived immunosuppressive peptide in mouse models

**DOI:** 10.1186/1471-2172-14-51

**Published:** 2013-11-18

**Authors:** Martin Tolstrup, Claus Johansen, Lars Toft, Finn S Pedersen, Anne Funding, Shervin Bahrami, Lars Iversen, Lars Østergaard, Mogens Duch

**Affiliations:** 1 Department of Infectious Diseases, Aarhus University Hospital, Brendstrupgaardsvej 100, 8200 Aarhus, Denmark; 2 Department of Dermatology, Aarhus University Hospital, 8000 Aarhus, Denmark; 3 Department of Molecular Biology and Genetics, Aarhus University, 8000 Aarhus, Denmark; 4 SKAU Vaccines Aps, Åbogade 15, 8200 Aarhus, Denmark

## Abstract

**Background:**

Short dimeric or mulitmeric peptides derived from a highly conserved stretch of amino acids from gammaretroviral envelope proteins has been found to have immunosuppressive properties *in vitro*. Here we test the hypothesis that such immunosuppressive peptides may serve as immunomodulatory reagents for treatment of inflammatory disorders.

**Results:**

The anti-inflammatory effect of a synthetic retrovirus-derived immunosuppressive peptide of 17 amino acids was tested in two murine skin inflammation models, a TPA-induced acute toxic contact eczema model and an oxazolone-induced allergic contact dermatitis. Overall, mice (n = 24) treated with a topically applied cream containing the dimeric immunosuppressive peptide exhibited a reduction of 28.8% in ear thickness (range 20.1-42.5), whereas the application of a scrambled peptide dimer or a monomer of the immunosuppressive peptide remained without effect (p = 0.028). Furthermore, ear biopsies from mice treated with the dimeric immunosuppressive peptide showed a significant reduction in mRNA of the pro-inflammatory cytokines TNF-α, IL-17C, and IL-6 as well as the chemokine CXCL2 compared to mice treated with control peptides.

**Conclusion:**

Using two murine skin inflammation models, we show that an immunosuppressive retroviral peptide is capable of reducing inflammatory disorders. The results indicate that virus-derived immunosuppressive peptides capable of down-regulating several proinflammatory cytokines may represent a novel class of drugs for the treatment of excess inflammation.

## Background

Several inflammatory diseases are characterized by an unbalanced inflammation and an increased expression of inflammatory cytokines such as TNF-α, IL-6, IL-17A, IL-17C, IL-20, IL-22, IL-23, IFNγ and CXCL2. Especially TNF-α, IL-17A and IL-12/IL-23 are believed to play a key role in the pathogenesis of psoriasis, which is confirmed by the successful use of antibodies directed against these cytokines in the treatment of psoriasis [1-3]. A subset of these cytokines such as TNF-α, IL-17C, IL-6 and CXCL2) have also been implicated in sepsis [4-8].

**Figure 1:**
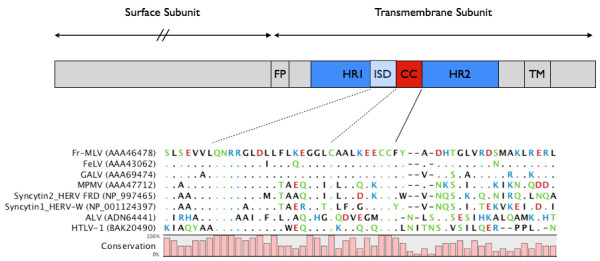
**Schematic representation of the retroviral envelope protein (not to scale).** Top figure displays some of the functional elements in the transmembrane subunit. The fusion peptide (FP) and the Heptad Repeat I/II (HR) followed by the membrane transversing region (TM). The Immunosuppressive Domain (ISD) is located just N-terminal of the highly conserved CC-motif forming a vital disulphide bond. Stipulated lines indicate amino acid position in the alignment. Fr-MLV Friend Murine Leukemia Virus; FeLV Feline Leukemia Virus; GALV Gibbon Ape Leukemia Virus; MPMV Mason-Pfizer Monkey Virus; ALV Avian Leukosis Virus; HTLV Human T-lymphotropic Virus. GeneBank accession numbers indicated in parenthesis.

It has been known for many years that retroviruses are capable of inhibiting proliferation of immune cells upon stimulation [9-11]. These effects have been localized to the viral fusion protein. Expression of such fusion proteins is sufficient to enable allogenic cells to grow into a tumor in immune competent mice [12]. Immunosuppressive (ISU) fusion proteins have been found in a variety of different viruses with type I fusion mechanism such as retroviruses like MPMV and HIV and in filoviruses such as Ebola and Marburg [13-17]. This ISU-activity was in all these cases located to a very well-defined structure in the transmembrane protein (Figure 1) the ISU effects range from significant inhibition of lymphocyte proliferation [14,15] cytokine skewing (up regulating IL-10; down regulating TNF-α, IL-12, IFN-γ) [18], inhibition of monocytic burst [19], polyclonal B cell activation and cytotoxic T cell activity [20]. Peptides spanning the ISU domain are as active as the whole protein in these assays but only when presented in a dimeric form or coupled to a carrier protein (i.e. >monomeric). Such multimeric peptides are capable of reducing or abolishing immune responses like cytokine secretion or proliferation of activated T-cells.

The ISU-domain has been investigated extensively using a 17-meric peptide (CKS-17) derived from murine leukemia virus (MLV) which shows significant immunosuppressive activity in vitro as described above [4,5,21-23].

The ability of CKS-17 to repress T-cell mediated immune responses makes this peptide an interesting drug candidate for the treatment of inflammatory diseases. The aim of this study was to verify the in vivo effects of a retrovirally derived CKS-17 peptide on the immune system. We used two known mouse models for skin inflammation: Acute allergic contact dermatitis model and TPA toxic eczema model [24,25].

In both models, the application of the peptide reduced the inflammation induced by the irritants, thereby confirming the viability of utilization of this virus-derived peptide for treatment of inflammatory diseases.

## Results

In order to retain the ISU-peptide on the ears of the mice for longer periods the dissolved peptide was mixed with Natusan®, First Touch Protection cream at a 1:1 ratio to create a homogenous solution. All peptides were freshly dissolved at the start of treatments and stored for 4°C during the animal experiments.

### Acute toxic contact eczema model

**Figure 2:**
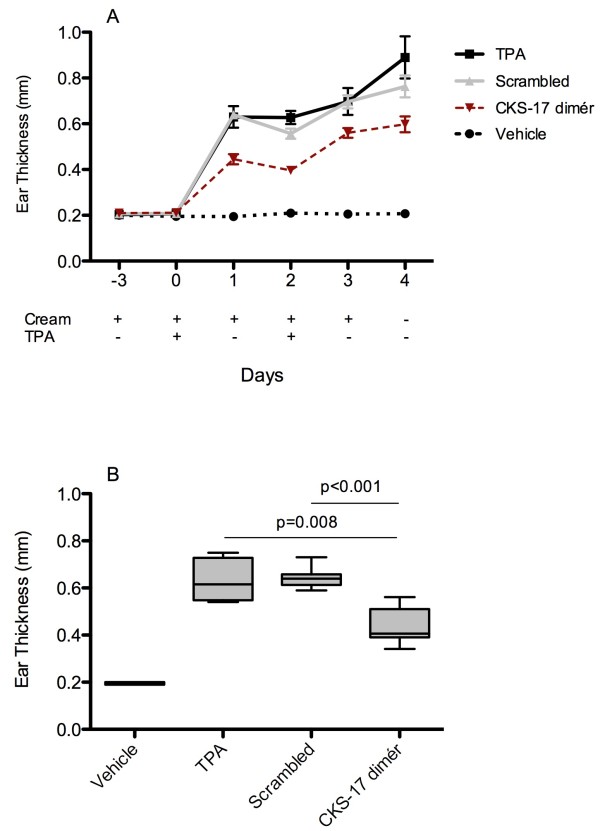
**Earthickness in an acute toxic contact eczema model (C57BL/6 mice). A)** Acute toxic contact eczema model in C57BL/6 mice (n = 6 pr group) pretreated with peptide cream prior to application of TPA. CKS-17 dimér and scrambled CKS-17 dimér used at a concentration of 100 uM. Ear thickness measured each day from onset of TPA treatment. The graph depicts mean with SEM. **B)** Box-plot with whiskers (5–95 percentile) of ear thickness measurement at day 1 after TPA application. Treatment group comparison performed by students t-test.

Two variants of the TPA-induced acute toxic contact eczema model were used to evaluate the anti-inflammatory potential of topical applied CKS-17 peptide. Initially, mice were pretreated for 3 days with peptide-containing cream followed by induction of ear inflammation by TPA. Figure 2A depicts the time-course. Evidently, mice treated with CKS-17 peptide have significantly reduced swelling of the ears upon inflammation induction. The direct comparison with a scrambled peptide in Figure 2B (day 1) points to a specific effect of CKS-17 dimer, which is significantly more potent than the scrambled peptide control to reduce ear inflammation (p < 0.001).

**Figure 3:**
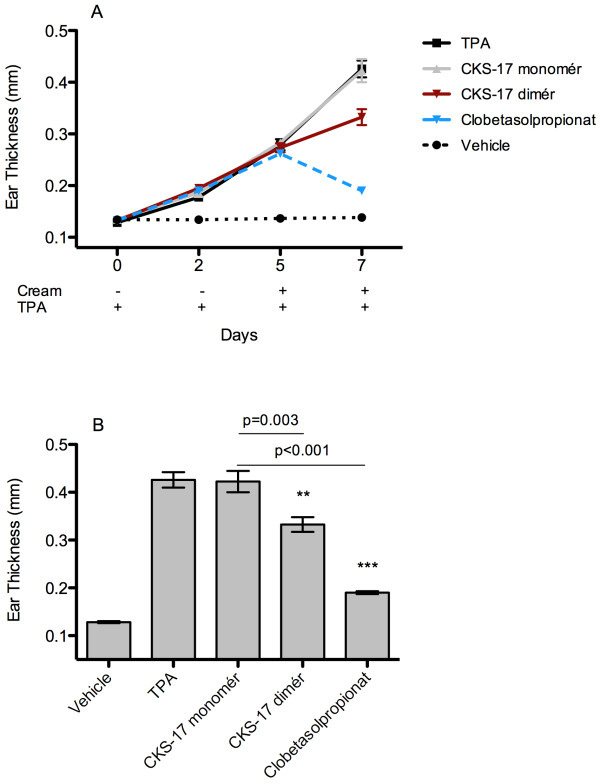
**Earthickness in an acute toxic contact eczema model (BALB/CJ mice). A)** Acute toxic contact eczema model in BALB/cJ mice (n = 6 pr group) with application of TPA at day 0 and treatment with peptide cream at day 5. CKS-17 dimér and CKS-17 monomér used at a concentration of 100 uM. The graph depicts mean with SEM. **B)** Box-plot with whiskers (5–95 percentile) of ear thickness measurement at day 7 after TPA application. Treatment group comparison performed by Student’s t-test. Clobetasol propionate 0.05% (GSK, UK) applied neat without dilution with PBS.

In the same model an experiment was performed in which inflammation was induced five days prior to treatment with peptide cream (Figure 3A). In this setup a monomer peptide vs a dimer was tested since the ISU-activity of the peptides are consistently correlated with di- or multimeric forms of the peptides [14,19,26]. This was confirmed in a direct comparison experiment, where CKS-17 dimer-containing cream significantly reduced ear inflammation in contrast to the monomeric peptide. Figure 3B clearly shows a significant reduction of inflammation two days after CKS-17 dimer treatment in contrast to the monomeric peptide (p = 0.003, Student’s t-test). Monomeric peptide was not different from the pure cream vehicle. Furthermore, in the same experiment we performed a direct comparison to a potent anti-inflammatory topical glucocorticoid based cream (Clobetasol propionate 0.05%). Clobetasol propionate treatment was very potent in this model and reduced inflammation close to the basal level with minimal inter-animal variation.

### Oxazolone-induced murine allergic contact dermatitis

**Figure 4:**
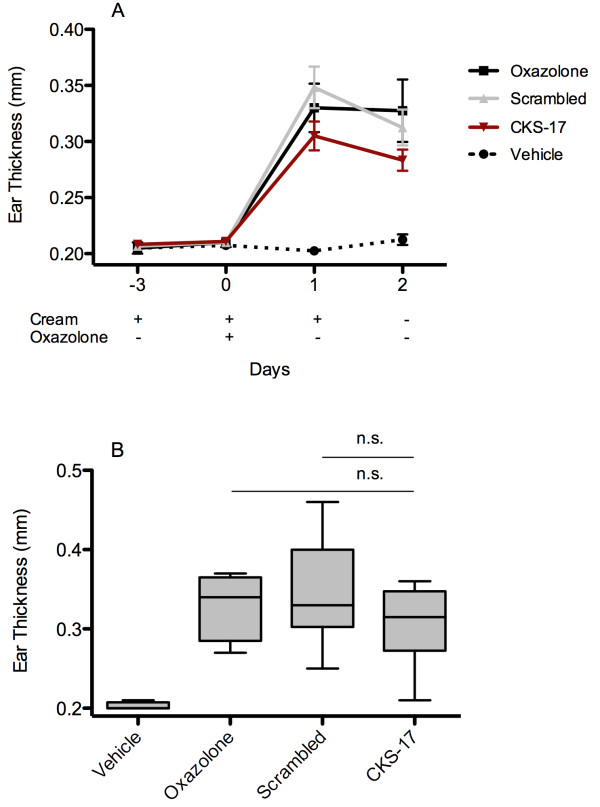
**Earthickness in an oxazolone-induced murine allergic contact dermatitis model. A)** Oxazolone-induced murine allergic contact dermatitis model in C57BL/6 mice (n = 6 pr group). At day −7 mice were sensitized by oxazolone on the abdomen. On day −3 ears were pretreated with cream followed by challenge by oxazolone on the ears at day 0. The graph depicts ear thickness mean with SEM. **B)** Box-plot with whiskers (5–95 percentile) of ear thickness measurement at day 2 after Oxazolone application. Treatment group comparison performed by Student’s t-test.

**Figure 5:**
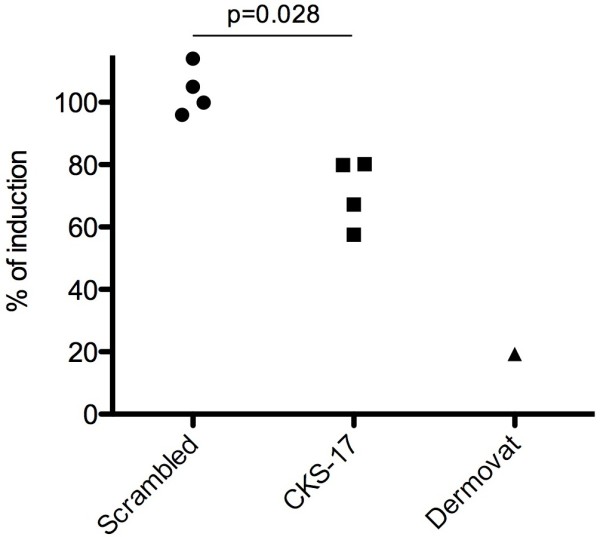
**Relative comparison of ear thickness across 4 independent studies.** Included in analysis: three studies in the acute toxic contact eczema model and one study in the oxazolone-induced murine allergic contact dermatitis model. Each point represents one experiment with the average relative ear thickness from each 6 mice. The relative induction calculated as [(ear thickness treatment two days post challenge - baseline ear thickness)/(ear thickness TPA/Oxazolone two days post challenge - baseline ear thickness)]*100%.

This model is representative of allergic contact dermatitis induced inflammation by sensitizing the mice 7 days prior to challenge. Three days before induction of inflammation the mice were treated with peptide-containing cream. Evidently, as in the TPA-induced eczema model treatment with CKS-17 containing cream lowered swelling of the ears (Figure 4A) following inflammation induction by oxazolone (day 0). However, the effect was less pronounced at day two post oxazolone challenge (p = 0.068 CKS-17 vs. scrambled, Student’s t-test) as shown in the box-plot in Figure 4B. In general, this contact dermatitis mouse model induces much less inflammation of the ears in comparison to the TPA mouse model. The results of a total of four independent experiments (3 eczema studies and 1 contact dermatitis) each with 6 mice pr treatment group are compiled in Figure 5. The figure depicts the relative reduction in ear thickness of the treatment groups (scrambled/monomer, CKS-17 or Clobetasol propionate) compared with TPA or Oxazolone alone. Across the experiments the reduction in the CKS-17 treatment group ranged from 20-43% with no effect of scrambled/monomer. Clobetasol propionate reduced the swelling of the ears by 80% in the single study where it was applied. The consistent reduction of treatment with CKS-17-containing cream independent of the model and scheme of application was significantly different from treatment with a scrambled peptide (p = 0.028, Mann Whitney test).

### The impact of CKS-17 treatment on cytokines implicated in inflammatory diseases

**Figure 6:**
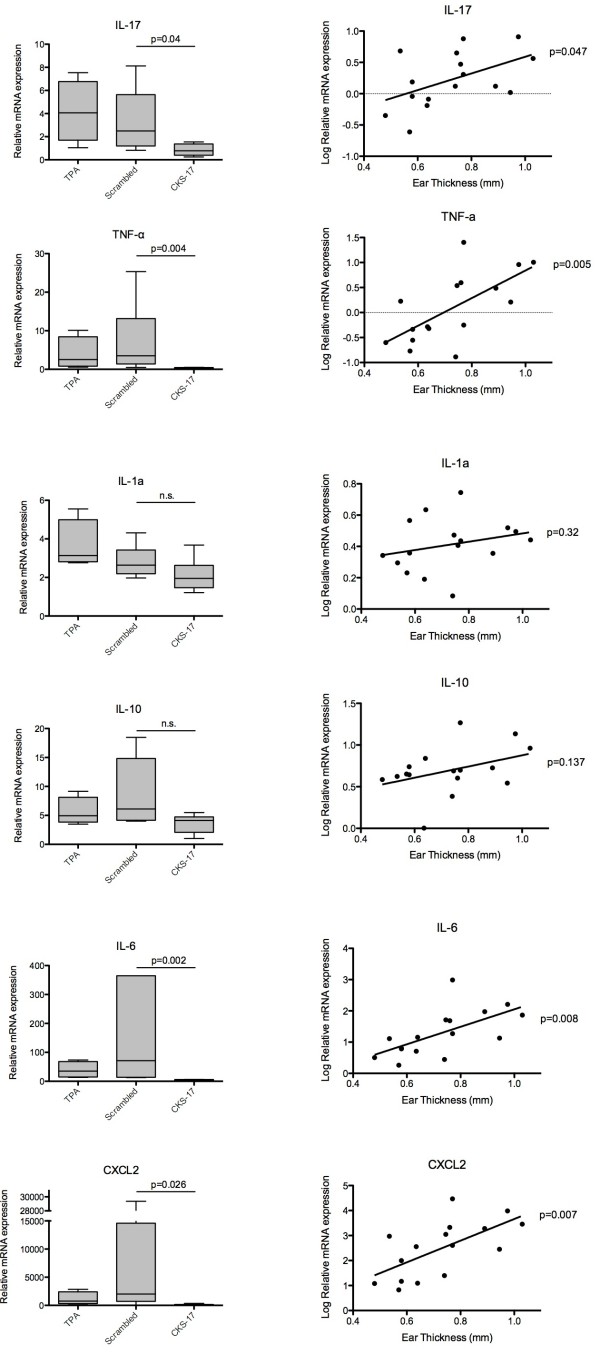
**Relative mRNA expression levels from ear biopsies of TPA treated mice.** Left columns depict Box-plot with whiskers (5–95 percentile) of the relative mRNA expression levels (Vehicle only mice index 1). On graphs statistical differences (Mann Whitney test) marked by p-value or as n.s. (non significant) between scrambled and CKS-17 treated mice. Right column depicts xy-plots of the Log_10_ normalized relative mRNA expression and ear thickness. Linear relationships were determined by Spearman correlation.

RNA from ear biopsies of the mice treated with TPA (mice depicted in Figure 2) was harvested and subjected to mRNA relative quantification of IL-1α, IL-6, IL-10, IL-17C, TNF-α and CXCL2. All expression levels were adjusted for input mRNA by GAPDH gene expression and the results normalized to levels for vehicle only treated mice. There was a clear and significant reduction of mRNA levels of IL-17C, TNF-α, IL-6 and CXCL2 in mice treated with CKS-17 containing cream (Figure 6, left column). When correlating the relative mRNA expression of these cytokines/chemokines to the ear thickness of individual animals, a statistically significant correlation was observed (Figure 6, right column). In turn, expression levels of neither IL-1α nor IL-10 were affected by treatment with CKS-17-containing cream compared to cream with scrambled peptide. Notably, plotting the expression levels of IL-1α and IL-10 against the ear thickness revealed no linear correlations where observed (Figure 6, right column). The results are summarized in Table 1.

**Table 1:** Cytokine mRNA expression index from ear biopsies

	**Vehicle**	**TPA**	**TPA w. scrambled**	**TPA w. CKS-17**	**p-value**
**IL-1a**	1(0.77–1.22)	3.64(2.76–5.55)	2.82(1.97–4.31)	2.1(1.21–3.67)	0.143
**IL-6**	1(0.81–1.19)	39.3(13.4–73.5)	217(12.9–976)	4.23(1.82–6.39)	0.068
**IL-10**	1(0.95–1.04)	5.63(3.49–9.16)	8.75(4.01–18.47)	3.61(1.00–5.49)	0.071
**IL-17c**	1(0.54–1.46)	4.17(1.04–7.54)	3.34(0.81–8.12)	0.85(0.24–1.54)	0.744
**TNF-α**	1(0.49–1.51)	3.93(0.56–10.1)	7.27(0.48–25.3)	0.3(0.17–0.52)	0.040
**CXCL2**	1(0.41–1.59)	1162(402–2846)	7339(12.3–29363)	86(6.7–358)	0.432

Interestingly, treating mouse ears with cream containing CKS-17 peptide results in a potent blockage of TNF-α mRNA expression (mean index (range) 0.3(0.17-0.52)) to levels below the background in untreated animals in the vehicle control group (mean index (range) 1(0.49-1.51)).

## Discussion

In this report we have tested the hypothesis that a retrovirus-derived immunosuppressive peptide may reduce inflammation. We found that a topically delivered retroviral derived peptide (CKS-17) reduced ear inflammation in two different mouse models. Neither a dimeric version of a scrambled ISU-peptide nor a monomer of the ISU-peptide had any effect, similar to what was previously reported in cell-culture-based assays [9,10,14,15,18-20,26]. The lowering of inflammation in the mouse models can be coupled to significantly reduced expression of key inflammatory cytokines like IL-6, TNF-α and IL-17C.

These results complement those of Cianciolo et al. who recently reported that the CKS-17 ISU-peptide or shorter versions thereof had an immunosuppressive effect in mouse models of inflammatory peritonitis and disseminated intravascular coagulation [27]. Parallel work on hydrophobic regions of HIV-1 gp41, both the fusion peptide and the ISU domain, has suggested control of T cell activation [17,28]. A different applicability of modifying the ISU-peptide from HIV-1 was reported by Morozov and colleagues who were able to enhance the vaccine induced IgG titer towards recombinant gp41 [29].

Previously, TPA-induced skin inflammation in mice has been shown to involve potent upregulation of TNF-α [30,31]. Here we report that TNF-α expression levels were the most severely affected cytokine level by peptide treatment. Despite a four-fold increase in mRNA by TPA treatment the application of CKS-17 containing cream reduced TNF-α expression to below baseline levels (average of 70% reduction compared to baseline). In addition, levels of another important regulator in autoimmune diseases, IL-17C was significantly reduced upon treatment of inflamed ears with cream containing retroviral peptide. Recently, anti-IL-17A receptor antibodies have proven successful in early clinical trials [2,30].

Interestingly, we find that the level of IL-1α expression is not regulated by CKS-17 peptide application. Considering the effect of IL-1α in the contact acute eczema model this was somewhat surprising although in agreement with earlier studies of the intrinsic effects of retrovirus-derived envelope peptides highlighting that CKS-17 does not block production of IL-1α but reduce the immunobiological effect [20]. The varied effect on several key cytokines suggests that the local application of peptide containing cream pursued in this study could be of greater relevance than systemic use of monoclonal antibodies mainly because of the plethora of targets and the fact that for example anti-TNF-α blockers carry increased infection risks [32].

In the model of Oxazolone induced inflammation a similar trend was observed in that ear inflammation was reduced in peptide treated mice. The degree of inflammation induced in this model is of a smaller magnitude. In turn, the relative reduction by peptide treatment was comparable to what was obtained in the TPA-induced model suggesting that the peptide did indeed impact this model as well, although the inhibitory effect failed to reach statistical significance. In this model we have previously shown mitogen-activated protein kinase AP (MAPKAP) kinase 2 regulated TNF-α as important for ear inflammation [24].

## Conclusion

We have shown that the immunosuppressive retroviral peptide CKS-17 is capable of reducing proinflammatory cytokines as well as the level of inflammation as measured by ear thickness in two mouse models of inflammatory skin disease. Together with the work of Cianciolo et al. [27] our results indicate that virus-derived immunosuppressive peptides capable of down-regulating several proinflammatory cytokines such as TNF-α, IL-6, and IL-17C may represent a novel class of drugs for the treatment of excess inflammation.

## Methods

### Peptides

CKS-17 peptide (LQNRRGLDLLFLKEGGLC) or scrambled CKS-17 (LGGEKLFLLDLGRRNQLC) both linked into dimers via the C-terminal cysteine were purchased as lyophilized powder from Anaspec (California, USA). CKS-17 monomer was purchased without the C-terminal disulphide linkage. All peptides were dissolved in sterile water.

### Mice

BALB/cJ and C57BL/6 mice were purchased from Taconic Europe (Ry, Denmark). All mice used in this study were female and 6–8 weeks of age when experiments were initiated. The animals were kept in animal facilities that maintained a temperature of 19–25°C, and a 12-hour day/night cycle. They were given access to food and water ad libitum. All experiments were approved by the Committee for Animal Experiments in Denmark.

### Acute toxic contact eczema model

Two different dosings were pursued in this model. Firstly, C57BL/6 mice were treated once daily from day −3 until day 3 on the ears with cream (commercially available baby lotion; Natusan®, First Touch Protection cream) + 50% H_2_O) alone, cream + scrambled peptide or cream + CKS-17 peptide. Before the first application of cream at day −3 the ear thickness was measured. At day 0 and day 2 (3 hours before treatment with cream) the mice were treated with phorbol 12-myristate 13-acetate (TPA)(Sigma-Aldrich, St Louis, MO, cat no. P8139) on the ears (2 μg/ear). TPA was dissolved in acetone. Before the first challenge of TPA the ear thickness was measured (day 0) and again post-challenge at the times indicated, using a Mitutyo digimatic indicator. In the second experiment, BALB/cJ were treated at day 0, 2, 5 and 7 with phorbol 12-myristate 13-acetate (TPA)(Sigma-Aldrich, St Louis, MO, cat no. P8139) on the ears (2 μg/ear). TPA was dissolved in acetone. Before the first challenge of TPA the ear thickness was measured (day 0) and again post-challenge at the times indicated, using a Mitutyo digimatic indicator. At day 5 and 7 the mice were treated one time with either cream alone, cream + CKS-17 monomér, cream + CKS-dimér or Clobetasolpropionat 0.05% cream formulation applied neat (GSK, UK). The mice were anaesthetised during the ear measurement.

### Oxazolone-induced murine allergic contact dermatitis

4-Ethoxymethylene-2-phenyl-2-oxazolin-5-one (oxazolone) was purchased from Sigma-Aldrich (cat no. E0753). Oxazolone was dissolved in acetone. At day −7 mice were sensitized with 1.5% oxazolone by application to the clipped abdomen (100 μl). Once daily from day −3 until day 1 the ears of the mice were treated with cream (commercial available baby lotion; Natusan®, First Touch Protection cream + 50% H_2_O) alone, cream + scrambled peptide or cream + CKS-17 peptide. Before the first application of cream at day −3 the ear thickness was measured. At day 0 the mice were challenged with 0.5% oxazolone at the ears (20 μl per ear). This was done 3 hours prior to treatment with the different cream formulations. Before challenge (day 0), the ear thickness was measured and again post-challenge at the times indicated, using a Mitutyo digimatic indicator.

### RNA isolation

At termination of one study of acute toxic contact eczema punch biopsies (4 mm) from mice ears were transferred to 1 ml of −80°C cold RNAlater-ICE (Ambion inc., Austin, TX). Samples were kept at −80°C until 24 hours before RNA purification at which time they were transferred to −20°C. On RNA purification, biopsies were removed from RNAlater-ICE and transferred to 175 μl of SV RNA lysis buffer added β-mercaptoethanol (SV Total RNA Isolation System; Promega, Madison, WI) and homogenized. RNA purification was completed according to the manufacturer’s instructions (SV Total RNA Isolation System; Promega). RNA was stored until further use at −80°C.

### Quantitative polymerase chain reaction (qPCR)

For reverse transcription, Taqman Reverse Transcription reagents (Applied Biosystems, Foster City, CA, U.S.A.) were used. For qPCR we used Platinum® qPCR SuperMix-UDG (Invitrogen, Carlsbad, CA, U.S.A.) with primers and probes were Taqman 20× Assays-On-Demand (FAM-labeled MGB-probes) gene expression assay mix (Applied Biosystems). Each sample was loaded as triplets and analyzed on a Rotorgene-3000 real-time PCR machine (Corbett Research, Cambridge, U.K.). Relative gene expression levels were determined by using the relative standard curve method as outlined in User Bulletin 2 (ABI PRISM 7700 sequencing detection system, Applied Biosystems). Briefly, a standard curve for each gene was made of fourfold serial dilutions of total RNA from a punch biopsy from the ears of mice. The curve was then used to calculate relative amounts of target mRNA in the samples. As housekeeping GAPDH was used. Assay ID for the primers and probes used in this study were as follows: IL-17c (Mm00439619_m1); TNF-α (Mm00443258_m1); IL-1α (Mm00439620_m1); IL-6 (Mm00446190_m1); IL-10 (Mm00439616_m1); CXCL2 (Mm00436450_m1); GAPDH (Mm99999915_g1).

### Statistics

Comparisons of ear thickness and relative mRNA levels between treatment groups were performed using a student’s t-test. Correlations between ear thickness and mRNA levels were determined using spearman correlations. Lastly, the percentage effect on reduction in ear thickness between scrambled and CKS-17 treated groups across four independent mouse experiments were compared using a Mann–Whitney test. All p-values reported are two-tailed and we set the significance level at 5%.

## Competing interests

The authors declare that they have no competing interests.

## Authors’ contribution

MT designed experiments, performed experiments, performed data analysis and wrote the manuscript. CJ designed experiments, performed experiments and wrote the manuscript, LT performed experiments. FSP wrote the manuscript, AF designed experiments, SB designed experiments, LI designed experiments, LØ designed experiments, MD designed experiments, performed data analysis and wrote the manuscript. All authors read and approved the final manuscript.
